# Editorial: Reviews in ARF, the most misunderstood G protein I ever knew: a collection of papers exploring the multifaceted functions of an ancient protein family

**DOI:** 10.3389/fmolb.2026.1831289

**Published:** 2026-04-01

**Authors:** Paul A. Randazzo, Lorraine Santy, Pei-Wen Chen

**Affiliations:** 1 Laboratory of Cellular and Molecular Biology, National Cancer Institute, Bethesda, MD, United States; 2 Molecular, Cellular and Integrative Biosciences Program, Pennsylvania State University, University Park, PA, United States; 3 Biology Department, Williams College, Williamstown, MA, United States

**Keywords:** ARF, ARL, GEF, GTPase, GTPase activating protein (GAP)

It has been over 45 years since Leonard Schleifer, a graduate student in Alfred Gilman’s lab, discovered a proteinaceous factor in mammalian cells that was necessary for cholera toxin to ADP-ribosylate the G protein Gs. After an initial publication (Schleifer, 1980; Schleifer et al., 1982), Richard Kahn picked up the project to purify the factor, ADP-ribosylation factor, Arf, to homogeneity (Kahn and Gilman, 1984). Shortly later, Rick found it bound guanine nucleotides (Kahn and Gilman, 1986). These seminal results led to decades of discovery. Arfs are part of a larger Arf family of 30 genes, including the Arf-like proteins, that have an evolutionary origin distinct from other small GTPases like Ras. The family affects a spectrum of cellular activities, from protein secretion, to maintenance of cilia, to cell migration, that are essential for eukaryote survival. Yet, with all that has been learned, cellular roles and fundamental mechanisms mediating the effects of Arf family proteins are still being discovered. Frontiers is excited to present this collection of papers, which nicely describes our current understanding of the Arf family, particularly where that understanding is lacking and alternative mechanistic hypotheses that may serve as a roadmap for discovery as the field moves forward.

Dejgaard and Presley provide a comprehensive review of our current understanding of regulation and function of the four Arfs that reside on the Golgi apparatus in “*Arfs on the Golgi: four conductors, one orchestra*.” The theme that runs throughout this review is the complexity of the Arf pathways. After a general overview of the Arf pathway, the review launches into a description of the class 1 and class 2 Arfs within the Arf clade in the family. Starting with Arf1, the authors emphasize the complexities of the pathway, with Arf1 found in multiple locations with disparate targets. As the authors go through exchange factors and GTPase-activating proteins on the Golgi apparatus, the lack of a single unifying mechanism becomes more apparent. The discussion of effectors is particularly enlightening with two Arf binding sites within coatomer and clathrin adaptors, the most studied effectors for Arfs. Two other papers in this collection discuss that Arf proteins do not appear to adhere to the mechanistic paradigms established for other GTPases, something inferred from this review.

Quirion et al.’s “*Unfolding Arf and Arl GTPases: from biophysics to systems-level insights*” focuses on the Arf-like proteins. The Arls affect lysosomes, microtubules, cilia and mitochondria. While sites of action and functions have been defined for specific Arls, the nucleotide cycling properties, the effectors and the regulation have not been well described, despite the fact that, unlike for Arfs, mutations in the Arls are associated with specific human diseases. After describing the Arf family, there is a brief discussion of the biophysics of Arf, which emphasizes that Arf is not like Ras. The authors introduce the Arls with the idea that at least some might not function as binary switches, referencing pseudoGTPases, a concept explored in the literature for Ras family proteins. Several example Arls are discussed, highlighting structural variations and differences in nucleotide binding properties that support the idea that some Arls have been found to fit the characteristics of pseudoGTPases. Two Arls are used as examples to go into further depth, Arl14 and Arl10. The authors emphasize that these are not binary switches like Ras and functional mutations in Ras may not (and probably do not) extrapolate in general to the Arls.

Turn et al. “*Arf, the most misunderstood G protein I ever knew: why study GAPs*” continues with the theme that paradigms for heterotrimer G proteins and Ras are poor models for the Arfs. Historical context is provided to explain the origin the binary switch model and why it was used to guide work on Arf. The authors then compare the properties of Arf to Ras, and explain from an evolutionary perspective considering Arf as part of the Ras superfamily is simplistic. The broader family containing the GTP binding proteins, the P-loop NTPases, is used as examples of proteins that cycle nucleoside tri and diphosphates but do not function as binary switches. The paper ends with discussion on how GTPase-activating proteins are essential to Arf function and speculation about how they might directly mediate effects of Arf. It is feasible that other Arf GAPs and GAPs for GTPases other than Arf might use similar mechanisms, opening another avenue of research based on this alternate paradigm.

The original research paper “*Loss of Arf5 impairs recovery after lysosomal damage*” by Iwaniec et al. nicely rounds the collection. Interestingly, although the evidence is compelling that Arf5 is involved in the important process of lysosomal repair, it did not support the obvious mechanistic hypothesis of protein recruitment, with Arf as the switch that targets the lipid transfer protein necessary for repair to the site of damage. The authors discuss that multiple Arfs with nonredundant roles might function at a single site, an idea that Dejgaard and Presley discuss for Golgi associated Arfs. While not addressed, exploring mechanisms beyond simple recruitment could offer valuable insights into lysosomal repair.

The four papers describe an ongoing journey of discovery of Arf proteins and their function. Although not completely misunderstood, mechanistic understanding of Arf and Arl function remains incomplete. We hope you enjoy the papers and, further, they will provide some guidance for further investigation into the function of Arfs.
